# Mechanisms of Action of the PGLYRP1/Tag7 Protein in Innate and Acquired Immunity

**DOI:** 10.32607/actanaturae.11102

**Published:** 2021

**Authors:** D. V. Yashin, L. P. Sashchenko, G. P. Georgiev

**Affiliations:** Institute of Gene Biology RAS, Moscow, 119334 Russia

**Keywords:** PGLYRP1/Tag7, cytotoxicity, antimicrobial effect, antitumor therapy, Mts1, Hsp70, HspBP1, TNFR1

## Abstract

One of the promising fields of modern molecular biology is the search for new proteins that regulate the various stages of the immune response and the investigation of the molecular mechanisms of action of these proteins. Such proteins include the multifunctional protein PGLYRP1/Tag7, belonging to the PGRP-S protein family, whose gene was discovered in mice at the Institute of Gene Biology, Russian Academy of Sciences, in 1996. PGLYRP1/Tag7 is classified as a protein of innate immunity; however, it can also participate in the regulation of acquired immunity mechanisms. In this paper, we consider the involvement of PGLYRP1/Tag7 in the triggering of antimicrobial defense mechanisms and formation of subsets of cytotoxic lymphocytes that kill tumor cells. The paper emphasizes that the multifaceted functional activity of Tag7 in the immune response has to do with its ability to interact with various proteins to form stable protein complexes. Hsp70-associated Tag7 can induce the death of tumor cells carrying the TNFR1 receptor. Tag7, associated with the Mts1 (S100A4) protein, can stimulate the migration of innate and adaptive immune cytotoxic lymphocytes to a lesion site. Involvement of Tag7 in the regulation of immunological processes suggests that it may be considered as a promising agent in cancer therapy. These properties of Tag7 were used to develop autologous vaccines that have passed the first and second phases of clinical trials in patients with end-stage melanoma and renal cancer. The C-terminal peptide of Tag7, isolated by limited proteolysis, was shown to protect the cartilage and bone tissue of the ankle joint in mice with induced autoimmune arthritis and may be a promising drug for suppressing the development of inflammatory processes.

## INTRODUCTION

Understanding the mechanisms of activation and action of regulatory and effector lymphocytes is necessary in order to identify the pathways of host defense against cells with foreign or modified antigens. Understanding these processes is important in anti-inflammatory and anticancer therapy. In this regard, one of the areas of focus in modern immunology is the investigation of the proteins involved in innate and adaptive immunity, which allows for a deeper understanding of the immune response principles and the causes of the dysfunctions in various pathologies. The search for new proteins that regulate the activity of the cells involved in the immune response and the investigation of the molecular mechanisms underlying the action of these proteins seem promising. PGLYRP1/Tag7 is one such protein.

The gene for this protein was discovered in mice by subtracting cDNA libraries obtained from metastatic and non-metastatic mouse tumor cell lines at the Institute of Gene Biology, Russian Academy of Sciences, in the laboratory headed by one of the authors (Georgy Pavlovich Georgiev), in 1996. The protein was given the working name Tag7 [1]. Tag7 turned out to be playing an important role in antitumor defense [2]: so, its name signifies the tumor antagonistic gene protein product.

In 1998, Kang *et al*. [3] found a gene in the insect hemolymph whose structure was highly homologous to that of the *tag7 *gene. The product of this gene was shown to bind to peptidoglycans of the bacterial cell wall and was termed the peptidoglycan recognition protein (PGRP) [3]. The structure of a mouse *PGRP* homologue is identical to that of the previously described* tag7 *gene [4, 5], which means that Tag7 and PGRP are the same protein. Later, when the gene family was discovered, the term PGRP was changed to PGRP-S (where S stands for “small”).

Further functional studies of Tag7/PGRP-S were conducted in two directions. While European and U.S. researchers have focused on the role of Tag7/PGRP-S in innate antimicrobial immunity, we have mainly studied the mechanisms of antitumor action of this protein and related issues (Institute of Biochemistry, Russian Academy of Sciences).

## PGRP/Tag7 PROTEIN FAMILY

The Tag7/PGRP-S protein belongs to a small protein family. Members of this family differ in their transcript lengths: extracellular PGRP-S (short form) [1, 3], long transmembrane PGRP-L [5, 6, 7], and intermediate PGRP-I [6]. Structural studies revealed a highly conserved region of 160 amino acid residues at the C-terminus of all the proteins of this family. This region contains three adjacent PGRP domains connected by segments with a conserved amino acid sequence [6]. Only PGRP-S has a signal peptide in front of the PGRP domain, indicating that PGRP-S can be secreted by the cell [5].

In humans, there are four PGRP family proteins designated as PGLYRP1, PGLYRP2, PGLYRP3, and PGLYRP4. The first one corresponds to Tag7/PGRP-S [8]. It consists of 196 amino acids and has a signal peptide and a PGRP domain. Its gene is expressed in the bone marrow, thymus, fetal liver, polymorphonuclear leukocytes, lymphoid cells of the duodenum, spleen, and lymph nodes, alveolar epithelium, and pulmonary endothelium [6, 9].

Analysis of the crystal structure of PGRP proteins revealed a ligand-binding site recognizing a specific peptidoglycan sequence. Also, there was a protein–protein recognition site formed by a unique hydrophobic groove and the conserved amino acid residues Leu65, Arg18, Thr90, Glu93, Phe94, and Leu133 [10].

The PGLYRP1/Tag7 structure determines its functional activity. The protein can participate in antimicrobial defense activation by binding to peptidoglycan. The protein–protein interaction sites are responsible for the association of PGLYRP1/Tag7 with other proteins, which is followed by the formation of the stable complexes involved in immune response triggering. PGLYRP1/Tag7 is usually referred to as an innate immunity protein, which is not entirely true (see below). Its involvement in the regulation of the immune defense has been extensively studied. There exist three main areas of investigation of the PGLYRP1/Tag7 functional activity: (1) participation of PGLYRP1/ Tag7 in antimicrobial defense; (2) role of Tag7 in human lymphocyte activation; (3) use of Tag7 in antitumor therapy. This article discusses in detail these areas characterizing PGLYRP1/Tag7 as an active immune response regulator.

## INVOLVEMENT OF PGRP FAMILY PROTEINS IN INNATE ANTIMICROBIAL IMMUNITY IN INSECTS

Insect PGRP proteins can induce an antimicrobial immune response through either the Toll receptor or the Imd pathway [11, 12, 13].

After peptidoglycan recognition, insect PGRP proteins interact with Grass serine protease that initiates a proteolytic cascade, leading to cleavage of the Spatzle protein. One of the resulting fragments, Spatzle, forms a homodimer, causing dimerization and activation of the Toll receptor that further induces an antimicrobial response [14]. The PGRP-L protein interacts with Imd upon activation of the Toll-independent immune response pathway. Imd, in turn, induces a second signaling pathway, also resulting in the secretion of antimicrobial peptides [11, 15, 16, 17].

## INVOLVEMENT OF PGRP FAMILY PROTEINS IN INNATE ANTIMICROBIAL IMMUNITY IN MAMMALS

All four PGRP family members in humans and other mammals are soluble secreted proteins possessing both recognition and effector functions [18, 19]. PGLYRP1, PGLYRP3, and PGLYRP4 can directly lyse both gram-positive and gram-negative bacteria [20, 21, 22, 23]. PGLYRP3 is a peptidoglycan amidase [24, 25].

Each of these proteins contains one or two PGRP domains with a binding site specific for a muramyl peptide fragment of bacterial peptidoglycan [18, 19]. In addition, PGRPs can interact with lipoteichoic acid and lipopolysaccharide [22, 26]. Thus, PGRPs interact with the entire outer membrane of gram-negative bacteria [27].

PGRP uses three cytotoxic mechanisms to lyse bacteria. Firstly, PGRP induces oxidative stress because of increased formation of hydrogen peroxide (H_2_O_2_) and hydroxyl radicals (HO•) [27, 28]. Secondly, PGRP triggers thiol stress, leading to the depletion of more than 90% of intracellular thiols. The third antibacterial effect is metal stress that results in increased concentrations of intracellular Zn^2+^ and Cu+ ions [27, 28]. Each stress response alone has only a bacteriostatic effect, while combined induction of all three stress responses simultaneously exerts a bactericidal effect [27].

The antimicrobial effect of PGRP is enhanced through cooperation with innate immune cells. For instance, during the phagocytosis of bacteria, phagocytic cells pump not only oxygen radicals, but also Cu^+^ and Zn^2+^ ions into phagolysosomes to enhance the antimicrobial effect [29, 30]. In response, bacteria increase the expression of Cu^+^ and Zn^2+^ ion exporters [27]. PGRP proteins prevent these changes by promoting bacterial lysis [28]. PGRPs were also shown to act synergistically with antimicrobial peptides [31]. Also, PGRP-S was shown to interact with the innate immune receptor TREM-1 that triggers a pro-inflammatory immune response. This interaction will be discussed below. Synergistic interaction with other host defense mechanisms further enhances the antimicrobial efficacy of PGRP and prevents the development of resistance, thus making PGRP an important component of the innate antimicrobial immunity.

## THE Hsp70–PGLYRP1/Tag7 COMPLEX KILLS VARIOUS TUMOR CELL TYPES

The first studies showed that a conditioned medium of VMR-0 tumor cells transfected with a Tag7-encoding construct has a cytotoxic effect on VMR-0 cells. Antibodies to Tag7 neutralize this effect, indicating that Tag7 is cytotoxic [2].

However, another group of researchers demonstrated that PGRP-S expressed in *Escherichia coli *cells has no cytotoxic activity [4].

Later, Tag7 produced in a yeast system was also found to lack any toxic effect. However, it can form a stable equimolar complex with the major heat shock protein Hsp70, which is highly cytotoxic [32]. The Tag7–Hsp70 complex at a concentration of 10-10 M can induce cell death in a wide range of tumor cell lines.

Two Hsp70 domains are required to form a stable complex. Tag7 can bind to the peptide-binding domain of Hsp70 and even to the 14-mer peptide of this domain, which is located on the tumor cell surface and plays an essential role in NK cell activation [33]. However, complexes of Tag7 with these Hsp70 fragments show low cytotoxic activity, and the presence of the Hsp70 ATP-binding domain leads to the formation of a highly active cytotoxic complex [32].

COS-1 cells transfected with *tag7 *were shown to release the Tag7–Hsp70 complex, which kills tumor cells, into a conditioned medium. The complex is secreted via the Golgi apparatus [32]. Apparently, VMR-0 cells transfected with *tag7 *also secrete the Tag7–Hsp70 complex, which explains the Tag7-dependent cytotoxic activity of a conditioned medium of these cells.

An intratumoral injection of the Tag7–Hsp70 complex was shown to inhibit tumor growth. For instance, administration of the Tag7–Hsp70 complex to mice subcutaneously inoculated with aggressive M3 melanoma cells suppressed tumor growth and increased animals’ life span more than two-fold [34].

LAK cells obtained by 6-day cultivation with cytokine IL-2 released the Tag7–Hsp70 cytotoxic complex into a conditioned medium after incubation with target tumor cells. A Golgi apparatus inhibitor suppressed the secretion of this complex by lymphocytes [32].

## INTERACTION OF THE Tag7–Hsp70 CYTOTOXIC COMPLEX WITH THE TNFR1 RECEPTOR INDUCES INTRACELLULAR CELL DEATH SIGNALS

A detailed study of the cytotoxic effect of this complex showed that different cells in heterogeneous tumor cell cultures died at different time intervals, and that different cell death mechanisms were induced in the cells. The cells incubated with the Tag7–Hsp70 complex underwent apoptotic death 3 h after incubation, while RIP1 kinase-mediated necroptosis was activated in them only 20 h later [35].

Both cytolytic processes are induced upon interaction of Tag7–Hsp70 with the same cellular receptor, TNFR1, which is specific to cytokine TNF-α.

TNFR1 is a member of the death receptor family; it can induce alternative cytotoxic pathways of programmed cell death: caspase-dependent apoptosis and RIP1-kinase-dependent necroptosis [36, 37]. Necroptosis pathways are induced in tumor cells with suppressed caspase activity through any of the pathways [38].

Tag7–Hsp70 binds to TNFR1 on the plasma membrane of tumor cells and interacts with its extracellular domain (sTNFR1) both in solution and on an affinity column. Antibodies to TNFR1 suppress this process in all cases. The Tag7–Hsp70 complex may be considered as a new ligand for the TNFR1 receptor, which induces various apoptotic and necroptotic pathways in tumor cells [35].

In apoptotic cell death, the cytotoxic effect of the Tag7–Hsp70 complex has to do with sequential activation of caspase-8 and caspase-3. No intracellular apoptosis mechanisms involving mitochondria and caspase-9 are activated [35].

**Fig. 1:**
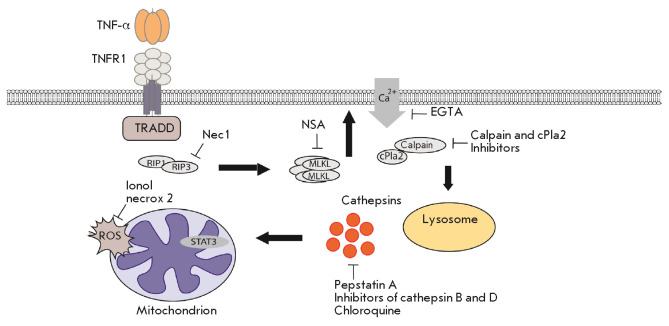
Schematic of necroptosis processes upon interaction of the Tag7–Hsp70 complex with the TNFR1 receptor on the surface of a target tumor cell

Necroptosis begins with necrosome formation, mediated by RIP1 and RIP3 kinases. The cytotoxic signal is further transmitted to cellular organelles: lysosomes and mitochondria. Accumulation of reactive oxygen species on mitochondrial membranes plays a key role in necroptotic cell death. There exists a relationship between lysosome activation and mitochondria. Inhibition of the catalytic activity of lysosomal cathepsins released into the intracellular space hinders both changes in the mitochondrial membrane potential and the accumulation of reactive oxygen species [39] (*Fig. 1*).

Tag7 and Hsp70 play different roles in the activation of cytotoxic pathways. Activation of a cytotoxic signal is known to be a two-stage process. At the first stage, a cytotoxic ligand binds to the receptor’s extracellular domain. At the second stage, the TNFR1 intracellular domain changes its structure to form the death domain that activates intracellular cytotoxic processes [40].

A necessary condition for death domain formation is the trimerization of the TNFR1 receptor [40]. In the absence of Hsp70, Tag7 is able to bind to TNFR1 as a monomer, but unable to induce receptor trimerization on the cell surface and, hence, trigger cell death. Tag7 inhibits the cytotoxic effect of both TNF-α and the Tag7–Hsp70 complex by competing with cytotoxic ligands for the TNFR1-binding site. Hsp70 cannot bind to TNFR1, but its interaction with Tag7 is necessary to induce cytotoxicity [35].

A 12-mer peptide at the Tag7 C-terminus was isolated by limited trypsinolysis. This peptide can bind to the TNFR1 receptor both in solution and on the cell surface [41]. The peptide was designated as “17.1” when first obtained by synthesis. Like the full-length Tag7, the 17.1 peptide did not induce cell death but inhibited the cytotoxic activity of both TNF-α and Tag7–sp70 [41]. Interestingly, the same peptide can interact with the heat shock protein Hsp70 and form the 17.1–sp70 cytotoxic complex that induces cell death [41].

The 17.1 peptide inhibited the functional activity of TNF-α not only in a cell model, but also in a mouse model. The anti-inflammatory effect of the 17.1 peptide was studied in a model of autoimmune arthritis induced by Freund’s complete adjuvant (FCA) stimulating tissue TNF-α secretion. This peptide was found to protect the cartilage and bone tissue of the ankle joint in mice [41]. We suggest that the 17.1 peptide may be a promising agent for preventing inflammatory processes.

## METASTATIC PROTEIN Mts1/S100A4 DESTROYS THE Tag7–Hsp70 COMPLEX

Many metastatic cancer cell lines are insensitive to the effect of Tag7–Hsp70. One of the key metastasis-stimulating proteins, metastasin 1 (Mts1/S100A4), belongs to the S100 family of Ca^2+^-binding proteins [42, 43]. Mts1 can form stable complexes with both Tag7 and Hsp70. Interestingly, Mts1 binds to the same region in the Tag7 protein as Hsp70. When Mts1 interacts with the Tag7–Hsp70 complex, the latter dissociates, with further formation of Mts1–Tag7 and Mts1–Hsp70 complexes that lack cytotoxicity [44]. Thus, Mts1 secretion by tumor cells protects them from the toxic effect of Tag7–Hsp70 [45].

Indeed, cells with a high Mts1 level are not targeted by the Tag7–Hsp70 cytotoxic complex. This complex usually induces a cytotoxic signal in tumor cells with a low metastatic potential [45]. Obviously, this is due to the secretion of high levels of the Mts1 protein by active metastatic cells, which leads to dissociation of the cytotoxic complex. Therefore, Mts1 secretion appears to be one of the ways for tumor cells to escape the action of cytotoxic agents.

## THE Tag7–Mts1 COMPLEX IS A CHEMOKINE

Investigation of Tag7 chemotactic activity has yielded contradictory data: some researchers have argued that Tag7 is unable to induce chemotaxis of lymphocytes [4], while others have found that neutrophil-secreted Tag7 is able to induce cell movement [2]. As in the case of Tag7 cytotoxicity, both research groups are partially right.

Tag7 lacks chemotactic activity, but the Tag7–Mts1 complex causes directed migration of NK cells and CD4^+^ and CD8^+^ T lymphocytes along the complex concentration gradient [46]. Interestingly, the Tag7– Mts1 complex has a number of features atypical of classical chemokines. Tag7–Mts1 is a two-component complex with a high molecular weight, and none of its constituent proteins possesses the Greek key structure typical of most chemokines. Nevertheless, this complex induces a chemotactic signal through the chemotactic receptor CCR5 specific for ligands with a classical chemokine structure [47].

Apparently, protein components of the Tag7–Mts1 complex play different roles in inducing chemotaxis. Mts1 can bind to the CCR5 extracellular domain and inhibit the interaction between this receptor and ligands. However, this binding is insufficient to induce cell migration. Tag7 cannot interact with the CCR5 receptor; however, it participates in the transduction of a chemotactic signal by binding to Mts1 [47].

The Tag7–Mts1 complex can be secreted by both innate and adaptive immune cells [46]. Interestingly, secretion of the Tag7–Mts1 complex and, hence, induction of directed lymphocyte migration occur without preliminary activation of immunocompetent cells. Hence, effector lymphocytes start migrating along the gradient of the Tag7–Mts1 complex concentration before the immune response onset, which provides a rapid immune reaction to pathogen invasion.

Thus, Mts1, on the one hand, destroys the Tag7– Hsp70 cytotoxic complex and, on the other hand, forms the Tag7–Mts1 complex recruiting different types of T lymphocytes to the tumor to attack tumor cells.

## Tag7 AND Mts1 PARTICIPATE IN THE ACTIVITY OF A NEW TYPE OF CD4+ LYMPHOCYTES DIRECTED AGAINST TUMOR CELLS LACKING HLA ANTIGENS

Tag7 and Mts1 also interact with each other in another process: the killing of tumor cells lacking the HLA complex by CD4^+^ lymphocytes.

CD4^+^ T lymphocytes are mostly immune regulatory cells involved in the activation of effector T cells by secreting a wide range of cytokines [48]. In addition, they can kill various cells, including tumor cells carrying major histocompatibility complex (MHC) class II proteins on their surface. Cell death is induced through the classical pathway by interaction between the TCR receptor and antigens in complex with MHC II, alongside with secretion of perforin and granzymes [48].

A new subset of cytotoxic CD4^+^ T lymphocytes has recently been identified. These lymphocytes kill tumor cells lacking MHC I and MHC II proteins but carrying the major heat shock protein Hsp70 on their surface [49].

IL-2 is shown to induce the generation of cytotoxic CD4^+^ and CD8^+^ T lymphocytes in LAK cells; these lymphocytes kill HLA- tumor cells upon interaction of FasL on the lymphocyte surface with the Fas receptor of target cells [50]. Tag7 is present on the plasma membrane of both subsets but has different functions.

Not only Tag7 and FasL but also Mts1 are present on the plasma membrane of cytotoxic CD4^+^ T lymphocytes. Mts1 is involved in the formation of an intercellular ternary complex between lymphocytic Tag7 and Mts1 proteins and Hsp70 on the target cell membrane. Along with Tag7, lymphocytic Mts1 is also required for the cytotoxic activity of these lymphocytes [44, 45].

**Fig. 2:**
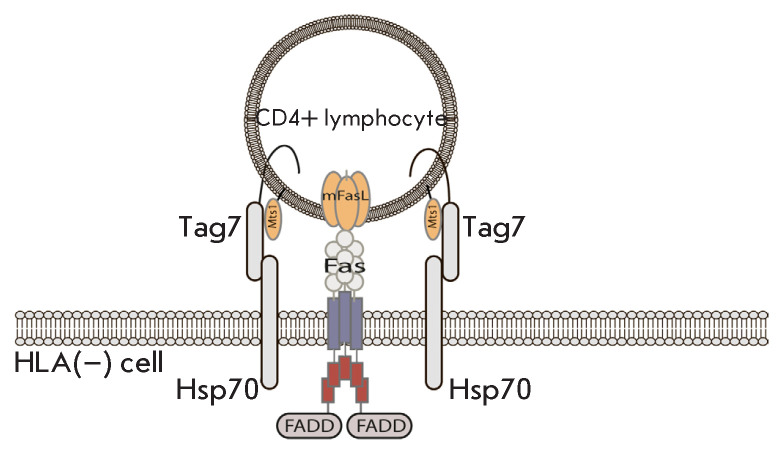
The Tag7–Mts1 complex is involved in the recognition of HLA-negative tumor cells by CD4^+^ cytotoxic lymphocytes

Thus, a sufficiently stable intercellular complex Tag7–Mts1–Hsp70 is formed, which allows the cytotoxic lymphocyte to anchor on the target cell surface. As a result, lymphocytic FasL interacts with the Fas receptor of the target cell and induces cell death. Both Tag7 and Mts1 are essential for cytotoxic activity [44] (*Fig. 2*).

Neither TCR nor granzymes are involved in the cytolysis of these cells; a cytotoxic signal is induced through the interaction between lymphocytic FasL and the Fas receptor of the target cell [49].

The CD127 antigen was detected on the surface of these CD4^+^ T lymphocytes, which is atypical of regulatory T cells (Treg) [50].

It is noteworthy that the described subset of T lymphocytes exposing the CD3, CD4, CD25, and CD127 antigens and Tag7, Mts1, and FasL proteins on their surface are present in the blood of healthy donors and accounts for about 1% of all T lymphocytes. Probably, the subset plays an essential role in fighting tumor cells that have lost their HLA complex during tumor progression.

## CD8+ T LYMPHOCYTES SECRETE THE Tag7–Hsp70 CYTOTOXIC COMPLEX

IL-2-activated CD8^+^ T cells can kill tumor cells that have lost surface antigens in a complex with MHC II and thus escaped the classical immune response. CD8^+^ T lymphocytes interact with these tumor cells via binding of the lymphocyte receptor NKG2D to the non-canonical MHC molecule MicA on the tumor cell. IL-2-activated CD8^+^ T lymphocytes form an intercellular NKG2D–MicA complex [51]. Although Tag7 is present on the membrane of these lymphocytes, and both MicA and Hsp70 are expressed on the membrane of the investigated tumor cells, no Tag7–Hsp70 complex forms between CD8^+^ T lymphocytes and target cells. This is probably due to the absence of Mts1 on the lymphocyte membrane (see above).

**Fig. 3:**
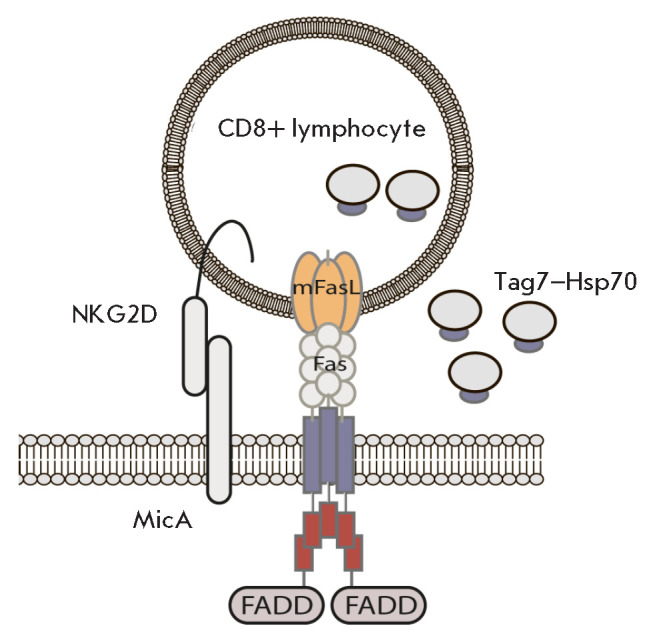
Schematic representation of the recognition and killing of HLA-negative tumor cells by a CD8^+^ cytotoxic lymphocyte. Contact with the target tumor cell leads to the secretion of the Tag–Hsp70 cytotoxic complex

The interaction of NKG2D with MicA underlies two of the activities of cytotoxic lymphocytes. Firstly, it is the induction of a cytotoxic signal, followed by the death of tumor target cells due to binding of the lymphocyte FasL to the Fas receptor of the tumor cell. Secondly, it is the secretion of a soluble Tag7–Hsp70 cytotoxic complex to the cell-cell contact area [52]. Binding of the Fas receptor to FasL on the lymphocyte surface is supposed to induce an accumulation of the Tag7–Hsp70 complex in the intracellular membranes of lymphocytes. Additional binding of MicA on the target cell to NKG2D on the lymphocyte surface is required for secretion of this complex, presumably for the formation of an intercellular contact area [52] (*Fig. 3*).

## HspBP1 CO-CHAPERONE IS INVOLVED IN THE REGULATION OF Tag7–Hsp70 CYTOTOXICITY

HspBP1 co-chaperone is an inhibitor of the ATPase activity of Hsp70; it can also bind to Tag7 and inhibit the cytotoxic activity of the Tag7–Hsp70 complex [53]. Various mechanisms can cause this. For instance, HspBP1 can bind to Tag7 and Hsp70, thus forming a ternary complex, followed by irreversible aggregation and formation of large conglomerates lacking cytotoxic activity. In addition, HspBP1 can competitively displace Hsp70 from the Tag7–Hsp70 complex. The resulting Tag7–HspBP1 complex has no cytotoxic effect on tumor cells. This complex is quite stable; it has been found in conditioned media of some tumor cells and human serum [54]. In the presence of high Hsp70 concentrations, Tag7–HspBP1 dissociates, with further formation of the Tag7–Hsp70 cytotoxic complex [53].

Interestingly, cytotoxic CD8^+^ T lymphocytes secrete Tag7–Hsp70 simultaneously with its inhibitor HspBP1; the cytotoxic activity of this complex persists for no more than 30 h. Addition of HspBP1 antibodies prevents inactivation of the secreted Tag7–Hsp70 complex during storage [53].

Thus, the inhibitor is present in lymphocytes containing the Tag7–Hsp70 complex and is secreted via the same mechanisms. Induction of its secretion also requires the formation of a contact area between the lymphocyte and the target cell [53].

## PGLYRP1/Tag7 BINDS TO THE TREM-1 RECEPTOR AND INDUCES MECHANISMS OF INNATE AND ACQUIRED IMMUNITY

It has been recently established that Tag7 is a ligand for the innate immunity receptor TREM-1 that belongs to the immunoglobulin superfamily and is expressed on monocytes and neutrophils [55]. TREM-1 is believed to be involved in the activation of monocytes and the pro-inflammatory immune response [56]. The interaction between Tag7 and TREM-1 leads to the activation of the genes encoding pro-inflammatory cytokines (TNF-α, IL-6, and IL-1β) and the secretion of their products [55, 57]. This is most likely one of the ways of Tag7 involvement in antimicrobial defense in mammals, which is associated with the secretion of these cytokines.

However, lymphocyte activation, followed by cytokine secretion, observed during the interaction between Tag7 and TREM-1 is not limited to the stimulation of antimicrobial defense mechanisms solely. An activation signal induced by the innate immunity protein Tag7 is transmitted to adaptive immune regulatory and effector lymphocytes and further promotes the formation of subsets of cytotoxic lymphocytes, killing tumor and virus-infected cells that have escaped immune surveillance [57]. As in the case of IL-2-activated lymphocytes, Tag7-activated CD4^+^ and CD8^+^ T cells were shown to recognize stress proteins (Hsp70 and the non-canonical molecules HLA and MicA) on the target cell surface and kill these cells through the FasL–Fas interaction via either apoptosis or necroptosis.

A low-molecular-weight immunity activator, Tilorone, was shown to induce production of the same cytotoxic lymphocytes, which indicates a common mechanism for the formation of these cytotoxic populations [58].

## Tag7 AND CANCER THERAPY

The data herein suggest that Tag7 is a promising anticancer agent. In fact, studies in this area have already been started. The very first studies on Tag7 functions assessed its effect on the growth of grafted VMR-0 tumors in mice [59, 60]. Like the vast majority of tumors, these cells do not synthesize Tag7. The cells were transfected, with genetic constructs providing a moderate expression of *tag7 *since its more active production results in cell death.

Control VMR-0 tumors grew rapidly and caused the death of mice after about a month. Tumors expressing Tag7 grew much more slowly and disappeared after several months. Next, the mice were administered a mixture of control and transfected cells. Growth of these tumors was intermediate between the growth of control and transfected cells. However, the tumors disappeared again after several months. Interestingly, Tag7-producing tumors were heavily infiltrated with NK cells, in contrast to the control tumors.

Given the obtained results, the first-phase clinical trials of autologous vaccines based on *tag7 *were carried out at the N.N. Blokhin Russian Cancer Research Centre (Moscow) and N.N. Petrov Research Institute of Oncology (St. Petersburg) [61, 62]. The trials were carried out in patients with either stage IV melanoma or stage IV renal cancer for whom all mandatory therapies had failed. Cell cultures were obtained from surgical samples. The cells were transfected with a construct carrying the human *tag7 *gene expressing the Tag7 protein. Following inactivation of the cells by X-ray irradiation, they were subcutaneously injected to the same patient from whom the tumor was obtained. The vaccine was shown to be completely safe; some positive effect was noted in 20–25% of cases, which was observed in the form of either tumor growth stabilization or its partial regression up to a complete reduction of large metastases.

Phase 2 clinical trials of these vaccines were carried out in 80 patients with the same tumor types at the N.N. Petrov Research Institute of Oncology [63]. The number of vaccine injections was increased (up to 26 injections). Some of the patients did not respond to therapy. Contact with the remaining patients was lost at different time points for reasons unrelated to the disease. Only those patients who were followed up for up to five years were taken into account. A total of 12 out of these 74 patients survived for more than five years: Contact with them was lost after 5–15 years. Moreover, the patients had no signs of tumor progression at the time of the last follow-up. The *Table* shows that the fate of some patients can be followed up for up to 15 years. Unfortunately, these results were not formally approved, because the trials were carried out according to the previous regulations, when preclinical studies were performed by a research and development laboratory, and vaccines were prepared not at a certified institution but either in a laboratory or in a clinic.

**Table:** Tag7 therapeutic effect

Tumor staging	Last follow-up (years after therapy)	Tumor progression	Baseline age
MELANOMA (63)			
3	15.4	No cases	33
3	15.2	Same	39
4	14.9	«–»	40
3	12.1	«–»	67
4	8.9	«–»	56
4	8.8	«–»	65
3	8.6	«–»	59
4	7.1	«–»	62
3	6.9	«–»	41
3	5.3	«–»	35
RENAL CANCER (11)			
4	9.9	There were no cases. The patient died 10 years later due to another cause	58
4	5.2	No cases	65

Complete cure of 16% of fatal patients is of certain interest, especially because there are suggestions as to why other cases failed. On the one hand, one of the most important factors in the described therapeutic approach seems to be the recruitment of different T lymphocyte types to the tumor. On the other hand, a number of mechanisms are known through which tumor cells become unrecognizable to protective T lymphocytes [64]. One of the important mechanisms is synthesis of DP-L1, a DP1 receptor ligand, by tumor cells [64]. Antibodies to DP-L1 or DP1 were shown to cause a strong therapeutic effect in patients with melanoma and other tumors, due to disrupted DP-L1– DP1 interaction [64]. There exist several commercial drugs of this type. A strong synergistic effect may be expected from a combined use of the two technologies, because each of them complements the other.

In addition, autologous vaccines should be substituted for allogeneic ones, which are much more technologically convenient. This switch requires a number of genetic, technological manipulations. There exist some studies in this area.

Thus, of 74 patients followed for ≥ 5 years (from the time of the last follow-up), 12 (16.2%) patients remained alive and had no signs of tumor progression at the last follow-up after > 5 years, while nine and three patients remained alive after seven and 15 years, respectively. The follow-up was terminated for reasons unrelated to the disease.

## CONCLUSIONS

In conclusion, we would like to emphasize that PGLYRP1/Tag7 is one of the key regulatory proteins involved in immune responses. PGLYRP1/Tag7 is classified as an innate immunity protein, but it can participate in the regulation of the immune mechanisms of both innate and acquired immunity. Tag7 induces antimicrobial defense mechanisms and the formation of subsets of cytotoxic lymphocytes killing cells that have escaped the antitumor immune response. The Tag7–Hsp70 complex causes the death of tumor cells carrying the TNFR1 receptor.

Investigation of the Tag7 crystal structure revealed the presence of a protein-protein interaction site in it. Apparently, Tag7 can interact with various proteins and this interaction determines its multiple functional activities. To date, the ability of proteins to change their function after interacting with other proteins and forming stable complexes is well known and referred to as moonlighting [65].

**Fig. 4:**
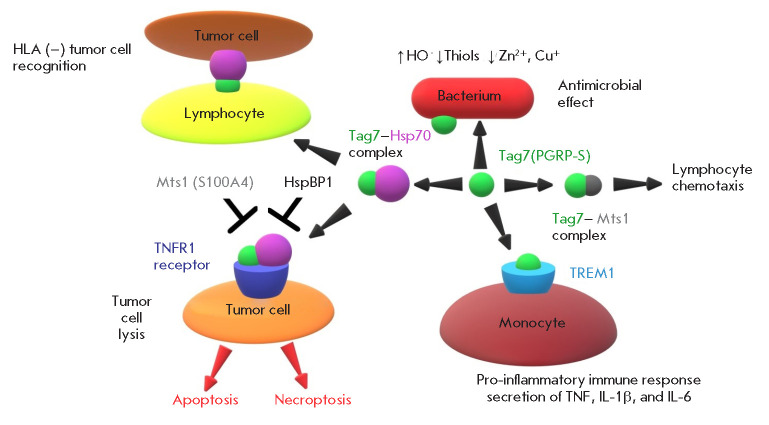
Functions of the PGLYRP1/Tag7 protein

The above data indicate that Tag7 can bind to five proteins: TREM-1, TNFR1, Hsp70, HspBP1, and Mts. Two of these proteins are receptors exposed on the plasma membrane of immune and tumor cells and involved in the induction of the immune response. The interaction of Tag7 with these proteins triggers the innate and adaptive responses involved in the host defense against pathogens (*Fig. 4*).

The antimicrobial effect of Tag7 in insects is associated with activation of the serine protease cascade, which converts Spatzle, a Toll receptor ligand, into an active form, followed by the release of antimicrobial peptides. The antimicrobial activity of PGRP in mammals is associated with three cytotoxic mechanisms: induction of oxidative, thiol, and metal stress. However, PGRP also functions in cooperation with other immune defense mechanisms and antimicrobial peptides.

The interaction between Tag7 and the human innate immune receptor TREM-1 at an early stage of monocyte activation results in the secretion of pro-inflammatory cytokines, inducing one of the antimicrobial defense pathways. Further transmission of the activation signal to regulatory cells activates subsets of cytotoxic lymphocytes, eliminating tumor and virus-containing cells that have lost their surface HLA antigens.

These lymphocytes can kill tumor cells through both the contact mechanism of lysis through the FasL–Fas interaction and the secretory mechanism through the release of the Tag7–Hsp70 cytotoxic complex into the contact area.

Secretion of the HspBP1 co-chaperone regulates the cytotoxic effect of the Tag7–Hsp70 complex. HspBP1 is secreted by lymphocytes simultaneously with the cytotoxic complex and can inhibit its activity through either disordered aggregation of the ternary Tag7– Hsp70–HspBP1 complex or dissociation of the Tag7– Hsp70 complex.

By binding to the extracellular domain of the receptor, Tag7 alone inhibits transduction of the cytotoxic signal to tumor cells. It is not only unable to cause cell death, but also inhibits the cytotoxic effect of other TNFR1 ligands, mainly TNF-α activity. Both Hsp70 and the formation of the cytotoxic complex on the cell surface are required for cytolysis induction by Tag7. In this regard, identification of a Tag7 peptide fragment modulating its functions is of particular interest. Expanding the spectrum of these functional peptides may be relevant in the development of drugs that inhibit acute inflammatory processes.

Involvement of Tag7 in the immune response is not limited to the activation of cytotoxic lymphocytes and the cytotoxic effect, together with Hsp70, on tumor cells. Tag7 can also interact with the Mts1 (S100A4) protein present in a wide range of metastatic tumors. Soluble Mts1 competes with Hsp70 for binding to Tag7, displacing the latter from the cytotoxic Tag7–Hsp70 complex to form an inactive Tag7–Mts1 complex. However, the Tag7–Mts1 complex has chemotactic activity and induces directed migration of innate and adaptive immune effector lymphocytes along the complex concentration gradient.

The Tag7–Mts1 complex is secreted by immune system cells, mainly neutrophils and monocytes, without pre-activation, which can yield rapid development of immune responses upon pathogen infection.

Experiments on mice using a number of tumor cell lines showed that an injection of tumor cells transfected with a construct producing Tag7 inhibits the growth of a grafted tumor of the same cell line. Autologous vaccines have been created based on these data; they have passed the first and second phases of clinical trials in fatal patients with melanoma or kidney cancer. Complete cure was observed in 12 out of 74 cases. There exist a number of opportunities to significantly improve the treatment effectiveness.

The above facts indicate that Tag7 is a multifunctional protein that is involved in the regulation of various stages of the immune response and is a promising agent for practical use in oncology.

## Abbreviations

Tag7: tumor antagonistic gene 7 protein product
PGRP: peptidoglycan recognition protein
FCA: Freud’s complete adjuvant
Mts1: metastasin 1
Hsp70: major 70-kDa heat shock protein
